# Synoptic reporting increases quality of upper gastrointestinal cancer pathology reports

**DOI:** 10.1007/s00428-019-02586-w

**Published:** 2019-05-29

**Authors:** Nikolaj S. Baranov, Iris D. Nagtegaal, Nicole C. T. van Grieken, Rob H. A. Verhoeven, Quirinus J. M. Voorham, Camiel Rosman, Rachel S. van der Post

**Affiliations:** 1 Department of Surgery, Radboud University Medical Center, Geert Grooteplein Zuid 10, PO Box 9101, 6500 HB Nijmegen, The Netherlands; 2 Department of Pathology, Radboud University Medical Center, Geert Grooteplein Zuid 10, PO Box 9101, 6500 HB Nijmegen, The Netherlands; 3 Department of Pathology, Cancer Center Amsterdam (CCA), Amsterdam UMC, VU University Medical Center, Amsterdam, The Netherlands; 4 Department of Research, Netherlands Comprehensive Cancer Organisation, Utrecht, The Netherlands; 5 PALGA Foundation (The Nationwide Network and Registry of Histo- and Cytopathology in the Netherlands), Randhoeve 225, 3995 GA Houten, The Netherlands

**Keywords:** Pathology report, Synoptic, Surgical pathology, Narrative

## Abstract

**Introduction:**

Traditionally, surgical pathology reports are narrative. These report types are prone to error and missing data; therefore, structured standardized reporting was introduced. However, the effect of synoptic reporting on the completeness of esophageal and gastric carcinoma pathology reports is not yet established.

**Materials and methods:**

A population-based retrospective nationwide cohort study in the Netherlands was conducted over a period of 2012–2016, utilizing the Netherlands Cancer Registry for patient data and the nationwide network and registry of histology for pathology data.

**Results:**

In total, 1148 narrative and 1311 synoptic pathology reports were included. Completeness was achieved in 56.4% of the narrative reports versus 97.0% of the synoptic reports (*p* < 0.01). Out of 21 standard items, 15 were significantly more frequently reported in synoptic reports.

**Conclusion:**

Synoptic reporting improves surgical pathology reporting quality and should be implemented in standard patient care.

**Electronic supplementary material:**

The online version of this article (10.1007/s00428-019-02586-w) contains supplementary material, which is available to authorized users.

## Introduction

Traditionally, surgical pathology reports are narrative, meaning they are written without a fixed form or structured outline [1]. Narrative reporting (NR), however, is prone to error, missing data, and inferior readability [2].

In 1991, the idea of a synoptic pathology format was introduced, meaning that completing a standardized structured form based on relevant and up-to-date guidelines will create a pathology report [1, 3]. Since its introduction, several studies have reported that synoptic or structured standardized reporting (SSR) significantly improves report completeness [4, 5]. Audits show that for esophageal carcinoma (EC) and gastric carcinoma (GC) narrative pathology reports completeness is an issue, in particular for resection margins and TNM stage [6]. Therefore, the College of American Pathologists’ esophageal and gastric oncology guidelines advise the use of SSR for pathology reporting [7, 8].

The main objective of this study is to assess the effect of SSR on the completeness of esophageal and gastric carcinoma pathology reports. We hypothesize that SSR will improve the completeness of reporting [9].

## Materials and methods

### Participants

A population-based retrospective nationwide cohort study was conducted including surgically treated patients with primary gastric or esophageal cancer, diagnosed between January 2012 and January 2016. Patient data, as registered by the Netherlands Cancer Registry (NCR), were linked to corresponding pathology report data from the nationwide network and registry of histo- and cytopathology in the Netherlands (*Pathologisch-Anatomisch Geautomatiseerd Landelijk Archief*; PALGA) [10]. Since not whole pathology reports, but only conclusion texts are available for research purposes via PALGA, we combined PALGA data with NCR patient data to ensure completeness of our data. All data has been anonymized.

Surgical pathology reports concerning EC and GC types 15.0 to 15.9 and 16.0 to 16.9 respectively, according to the 10th edition of International Classification of Disease (ICD), were included in the study. The period 2014–2016 was selected, since both SSR and NR were used during that period, with SSR for EC and GC being gradually introduced in March 2014 in the Netherlands. Narrative pathology reports from the cohorts 2012 and 2013 were added as a separate reference group. The small number of SSR in this period of introduction (*n* = 45) were not included in further analyses.

Exclusion criteria were endomucosal excisions, more than one primary tumor described, malignancy primarily originating from other tissue than esophageal or gastric tissue, and biopsy, i.e., non-surgical, reports.

### Primary outcome

The primary outcome was completeness, which was defined as the inclusion of all items in the pathology report as recommended by Dutch guidelines (www.oncoline.nl), comparable to the guidelines of the College of American Pathologists’ (CAP) [7, 8]. Standard items for EC, to be present in pathology reports are “histological type,” “differentiation grade,” “invasion depth,” “proximal/distal/circumferential resection margin,” “total and metastatic number of lymph nodes,” “size,” “location,” and “tumor regression after neoadjuvant therapy.” Histological subtypes were based on the WHO classification and Lauren’s system [11, 12]. Standard items for GC were identical, except for circumferential margin, since this is not applicable for the stomach.

Differentiation grade is applied for grading squamous cell carcinomas and intestinal adenocarcinomas according to the WHO classification system [12]. The item “subtype of adenocarcinoma (AC)” was only analyzed in adenocarcinomas. “Perineural invasion,” “lymphovascular invasion,” and “subtype of AC” were regarded as optional items for both carcinomas.

### Secondary outcome

Completeness was also compared when pathology reports were further grouped into academic and non-academic centers regarding SSR.

### Statistics

Statistical analysis was performed with the SPSS software package, version 24.0 (SPSS Inc., IBM Corporation Software Group, Somers, NY, USA). For dichotomous or ordinal variables, the Pearson’s chi-squared test or Fisher’s exact test was used if appropriate. All tests were two-sided, and *p* values less than 0.05 were considered to be statistically significant.

## Results

**Fig. 1:**
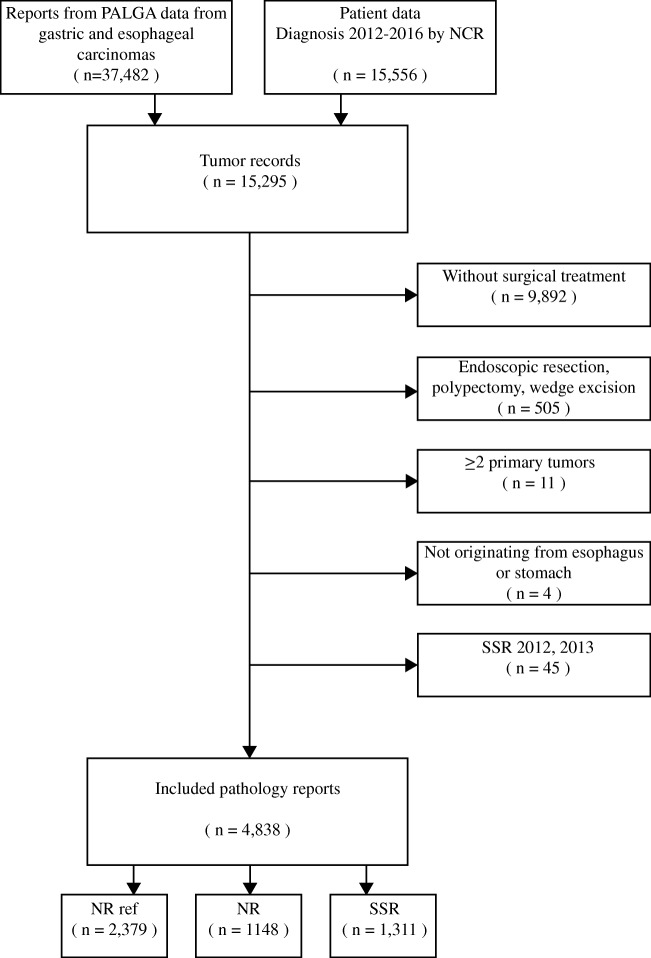
Flowchart depicting the selection process for eligibility. NR ref., narrative reports from the period 2012, 2013 used as reference; NR, narrative reports; SSR, structured standardized reports

In total, 4838 pathology reports were included: 2379 (49.2%) reference, 1311 (27.1%) synoptic and 1148 (23.7%) narrative reports (Fig. 1). Regarding SSR, there was no statistical difference between the academic and non-academic setting in complete reports for either EC or GC (both *p* = 0.80).

### EC completeness (Table 1 and Fig. 2)

In total, 2858 EC pathology reports were included, 685 narrative and 806 synoptic and 1367 in the reference cohort. Completion was achieved in 450 (65.7%) narrative versus 789 (97.9%) synoptic reports (*p* < 0.01). Besides “number of lymph nodes” and “T stage,” which were comparable, all standard items were significantly more frequently reported in SSR. The difference in completeness between SSR and NR did not affect the distribution of reported histological tumor types in the EC group (*p* = 0.46): adenocarcinoma 890 (80.8%), squamous cell carcinoma 154 (14.0%), and other types 57 (5.2%). Regarding optional items, SSR resulted in a much higher percentage of reported “lymphovascular invasion” and “perineural invasion”.

**Table 1:** Completeness and standard items

	Esophageal carcinoma				Gastric carcinoma			
	Reference *N* = 1367	NR *N* = 685 (%)	SSR *N* = 806 (%)	*p* value****	Reference *N* = 1012	NR *N* = 463 (%)	SSR *N* = 505 (%)	*p* value****
Overall completeness	666 (48.7)	450 (65.7)	789 (97.9)	< 0.01	349 (34.5)	197 (42.5)	483 (95.6)	< 0.01
T stage	1338 (97.9)	678 (99.0)	804 (99.8)	0.09	1007 (99.5)	459 (99.1)	505 (100)	0.05
Number of lymph nodes*	1365 (99.9)	685 (100)	806 (100)	–	1005 (99.3)	459 (99.1)	505 (100)	0.05
Histology type**	855 (97.9)	391 (97.5)	276 (100)	0.01	929 (98.5)	412 (99.0)	416 (99.8)	0.22
Subtype of AC^1^	315 (36.8)	194 (49.6)	149 (54.0)	0.27	668 (71.9)	327 (79.4)	399 (95.9)	< 0.01
Differentiation grade***	159 (72.3)	122 (84.1)	119 (97.5)	< 0.01	214 (81.7)	125 (89.3)	206 (100)	< 0.01

AC, adenocarcinoma

*Only “number of lymph nodes” are reported, since the “number of positive lymph nodes” resulted in exactly the same numbers

**Excluded were reports describing T0 and Tis carcinomas and subtotal regression after neoadjuvant therapy

***Included were reports describing squamous cell carcinoma or adenocarcinoma intestinal type

****NR versus SSR

**Fig. 2:**
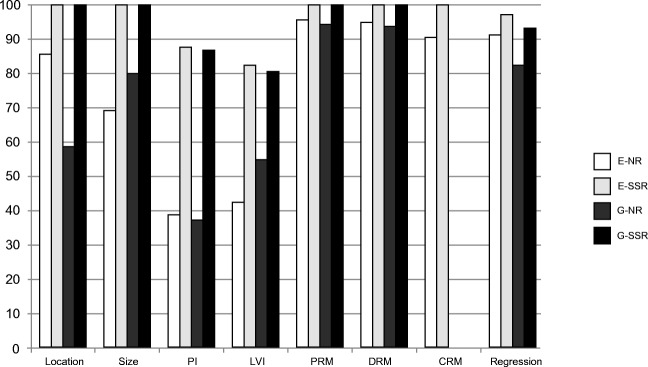
Recommended and optional items. LVI, lymphovascular invasion; PI, perineural invasion; PRM, proximal resection margin; DRM, distal resection margin; CRM, circumferential resection margin; regression, tumor regression after neoadjuvant therapy; E-NR, esophageal narrative reporting; E-SSR, esophageal structured standardized reporting; G-NR, gastric narrative reporting; G-SSR, gastric structured standardized reporting

### GC completeness (Table 1 and Fig. 2)

In total, 1980 GC pathology reports were included, of which 463 narrative, 505 synoptic and 1012 in the reference cohort. In total, 197 (42.5%) NR were complete versus 483 (95.6%) SSR (*p* < 0.01). “Differentiation grade” was significantly more reported in SSR compared with NR (100% versus 89.3%, *p* < 0.01), whereas “invasion depth,” “number of lymph nodes,” and “histological tumor type” showed no significant difference between NR and SSR. Reporting of GC histological tumor type, consisting mainly of adenocarcinoma (864 (96.0%)), did not differ between SSR and NR; however, “subtype of AC” was less frequently reported with NR compared with SSR (79.4% versus 95.9%, *p* < 0.01). The optional items, “lymphovascular invasion” and “perineural invasion” were much more frequently reported with SSR.

#### Missing data

Missing data in SSR was caused on one hand by software-related issues in the early stages of implementation (teething problems). For EC, 20%, and GC, 19% of the omitted items were due to software-related factors. Also, in the SSR software, differentiation grade is considered optional when neoadjuvant therapy was given (since grading is not possible in (sub-)total regression). On the other hand, the option of manually turning off several features, including standard items, remained through all development stages. It remains up to the pathologist whether he uses the complete protocol and/or the standard pathology conclusion or not.

## Discussion

This study shows that SSR increases the completeness of surgical esophageal and gastric cancer resection pathology reports, in particular for items as tumor size, grade, location, tumor regression after neoadjuvant therapy. SSR also increases lymphovascular invasion and perineural invasion reporting. Essential items for staging including depth of invasion, number of (metastasized) lymph nodes and tumor type were present in almost all narrative reports, and only minimal improvements could be achieved by SSR. For GC reporting especially, SSR leads to an increase in the reporting of histological subtype of adenocarcinomas. This is clinically relevant, since poorly cohesive adenocarcinomas (WHO) show a different biological behavior resulting in different clinical patient outcomes [13].

The differences between NR and SSR, regarding pathology report completeness, are in line with the systematic review by Sluijter et al. [1]. In accordance with Messenger et al., ours show no difference between the non-academic and academic care setting, looking at the effect of SSR on report completeness [4], implying that report quality much less depends on the pathologist’s specialization and, therefore, seems to help *all* pathologists with their histopathology reports. Although improved report completeness does not seem to have a direct impact on adjuvant treatment in GC and EC, as is the case in colorectal cancer—since the presence of lymph node metastases and pT4 tumor stage necessitate adjuvant therapy—this might very well change in the future. We do not claim that the introduction of SSR is the only or main factor for improved pathology reporting. Other factors, that may have influenced awareness among Dutch pathologists for EC/GC pathology reporting, are increased centralization of gastro-esophageal oncological surgery, comparative feedback by the Dutch Upper GI Cancer Audit (DUCA) (https://dica.nl/duca/) and monitoring during the multicenter gastric cancer CRITICS trial [14].

To our knowledge, this is the first report on a nationwide level with roughly 5000 pathology reports describing the influence of SSR on the completeness of EC/GC surgical pathology reports. The data originate from a nationwide database, which increases the power of the overall study, despite its retrospective nature.

Furthermore, PALGA’s SSR software is considered to be level 6 on the 1–6 reporting level and quality scale developed by Shrigley et al., which scores pathology reporting on format, content and several other quality indicators [15]. Level 1 corresponds to the traditional narrative report with a single text field, manual abstraction and a report completion duration within months to years after clinical procedure and level 6 to the standardized structured report with common data and messaging standards, discrete data fields, automated abstraction and report completion within weeks after clinical procedure. The fact that PALGA-software is considered level 6 makes it reasonable to suggest that our results can at least partly be attributed to the effect of using SSR [15].

Despite comparing reporting type on academic and non-academic level, inter- and intra-pathologist reporting differences were not accounted for, which possibly could have affected the completeness of reports [4].

In conclusion, our study has shown that SSR definitely improves the quality of surgical pathology reports of EC/GC and, therefore, should be part of standard patient care.

## Electronic supplementary material

ESM 1 (PDF 354 kb)
