# Supplementary figures and images for: Synoptic reporting increases quality of upper gastrointestinal cancer pathology reports
Source: Virchows Arch. 2019 May 29;475(2):255–9. doi: 10.1007/s00428-019-02586-w (PMC6647878; doi:10.1007/s00428-019-02586-w)

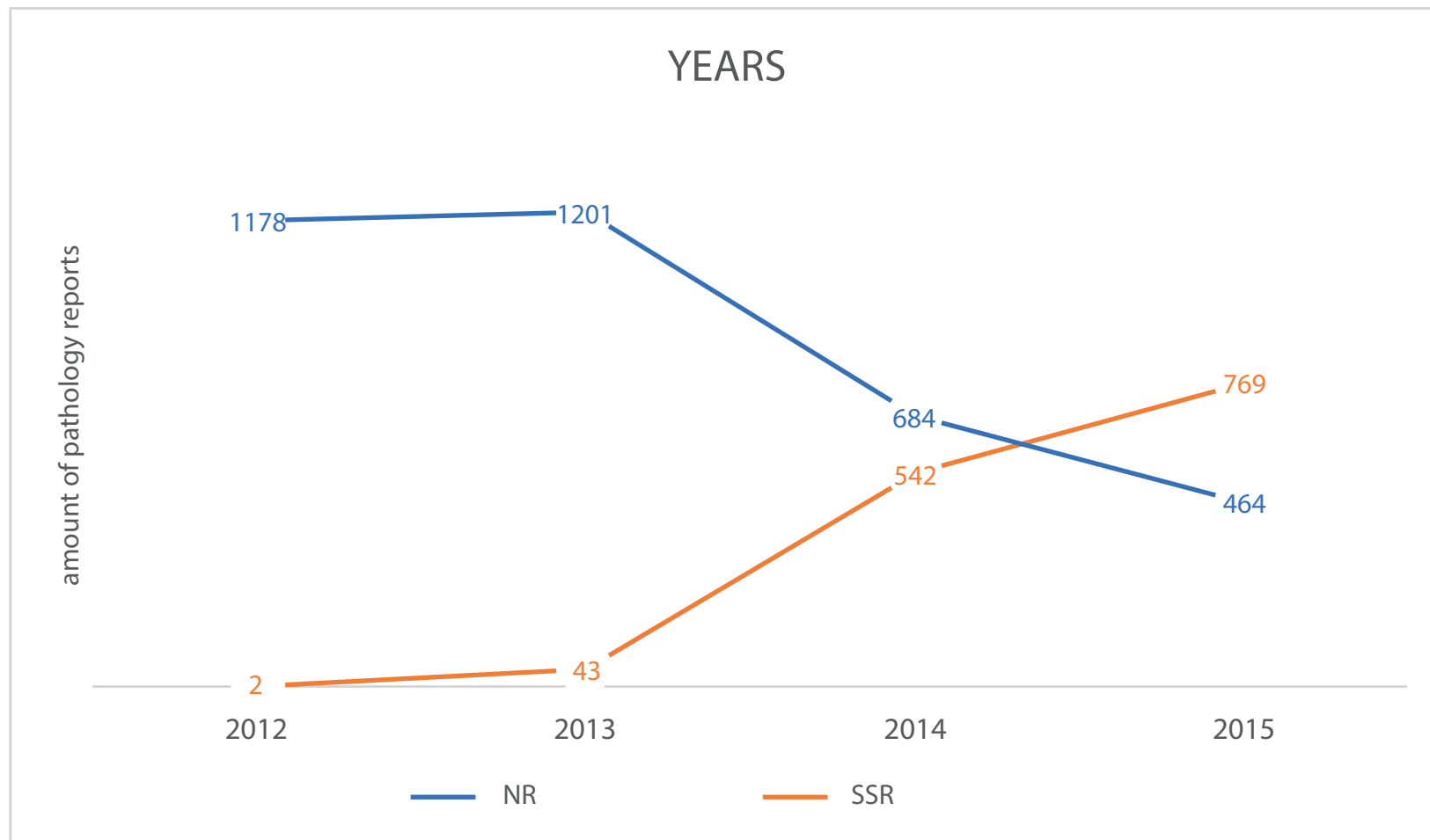

Supplement: Supplementary file 1 — (PDF 354 kb) [file 428_2019_2586_MOESM1_ESM.pdf]
